# Clinical course and perinatal management of fetuses and newborns affected by trisomy 13 and 18: a retrospective single-centre cohort study

**DOI:** 10.1007/s00431-025-06614-7

**Published:** 2025-12-20

**Authors:** Theresa Reischer, Tim Dorittke, Katrin Klebermaß-Schrehof, Angelika Berger, Paul Dremsek, Julia Ragossnig, Paula Höfinger, Gülen Yerlikaya-Schatten

**Affiliations:** 1 Department of Obstetrics and Gynaecology, Division of Obstetrics and Feto-Maternal Medicine, Medical University of Vienna, Vienna, Austria; 2 Department of Pediatrics and Adolescent Medicine, Division of Neonatology, Intensive Care and Neuropediatrics, Comprehensive Center for Pediatrics, Medical University of Vienna, Vienna, Austria; 3 Center for Pathobiochemistry and Genetics, Medical University of Vienna, Vienna, Austria

**Keywords:** Trisomy 13 and 18, Termination of pregnancy, Survival time, Palliative care, Perinatal management

## Abstract

Objectives Trisomy 13 (T13) and 18 (T18) are lethal chromosomal abnormalities. While the prognosis is generally poor, recent evidence suggests that some infants with T13 and T18 are able to survive longer than previously supposed. These findings underline the importance of an individualised approach to perinatal/neonatal care. Methods This retrospective study included 193 pregnancies with T13 and T18 in a single tertiary center between 2005 and 2023. Fetal and maternal characteristics, overall survival, and perinatal and postnatal management were analysed. Results Of 193 affected fetuses, 142 (73.6%) were diagnosed with T18 and 51 (26.4%) with T13. Associated malformations were present in 90.7% (175 cases), with congenital heart defects being the most common (42%). A total of 161 (83,4%) women opted for termination of pregnancy (TOP). Gestational age at diagnosis was significantly higher among women opting to continue pregnancy compared to those opting for TOP. Among women continuing pregnancy, 65.6% (21/32) experienced intrauterine fetal death. Eleven pregnancies resulted in live births. Survival times for infants born alive ranged from 6 min to 60 days, with a median survival time of 18 h. Newborns affected by T18 lived significantly longer than those with T13 (median 33 h vs 6.5 h).

*Conclusion*: The duration of survival in infants with T13 and T18 remains highly variable and difficult to predict. Management strategies must therefore be clarified within an interdisciplinary framework, supporting families through informed, individualised decision-making while balancing the poor prognosis with a focus on comfort, dignity, and quality of life.

**Supplementary Information:**

The online version contains supplementary material available at 10.1007/s00431-025-06614-7.

## Introduction

Trisomy 13 (T13) and trisomy 18 (T18) are severe chromosomal abnormalities associated with a range of serious and often fatal congenital defects. While the prognosis for affected individuals is generally poor, recent evidence suggests that some infants with trisomy T13 and T18 may survive for longer periods than previously supposed. Most live born children affected by these conditions die within the first days and weeks of life [1]. Average survival is described as 10–14 days, with survival beyond one year reported in 8–10% of cases [2, 3]. Some studies have even reported 5-year survival rates of 9.7% for T13 and 12.3% for T18, although at each timepoint, survival is consistently lower for children with T13 compared to T18 [4]. As a result of these findings, healthcare providers and families are increasingly faced with complex decisions regarding the management of affected pregnancies and neonates. In general, management focuses on providing supportive palliative care, addressing the specific medical needs of each individual child, such as respiratory and cardiac function support, or nutritional management. In some settings, surgical interventions may be considered to address particular congenital abnormalities [5]. However, at our institution, the approach has been to priorities comfort care and quality of life. Invasive procedures, such as mechanical ventilation and corrective surgery, have not been offered, in order to minimise unnecessary burden and suffering for these vulnerable patients. Although there have been reports advocating for more aggressive treatment strategies in view of longer-term survival [6], it remains essential to carefully balance these considerations against the overall poor prognosis and the ethical responsibility to avoid disproportionate interventions.

This paper provides an overview of the perinatal and postnatal management of pregnancies and neonates affected by T13 and T18 at our tertiary referral center. The aim of this study was to describe fetal and neonatal outcomes in affected pregnancies, to evaluate perinatal and postnatal management strategies and subsequently offer medical professionals and affected families evidence-based information to support informed decision-making, and to promote a multidisciplinary, family-centered approach to care.

## Methods

### Study population

A retrospective cohort study was conducted including 193 cases diagnosed with T13 and T18 between 2005 and 2023 at the Department of Obstetrics and Feto-maternal Medicine at the Medical University of Vienna. All pregnant women with an affected fetus received a detail anomaly scan at the time of referral, as well as regular ultrasound check-ups at our tertiary center, performed by a fetal medicine specialist. All cases were genetically confirmed following invasive testing including chorionic villi sampling (CVS), amniocentesis, and placental puncture, or postnatal genetic testing in a few cases. All patients were followed up until delivery and/or death of the infant. The options of termination of pregnancy (TOP) and primary palliative care management were discussed with all expectant parents. All cases in which TOP was considered were first discussed by an internal ethical committee. After 23 + 0 weeks of pregnancy (WoP), feticide was performed in most cases prior to induction. Depending on the severity of associated structural malformations, induction without feticide after 23 + 0 WoP was also offered. Labour was induced following a standard protocol with misoprostol after mifepristone. If women decided to continue pregnancy, a multidisciplinary team—including obstetricians, neonatologists, pediatric palliative care specialists and psychologists, provided counselling to the parents. During these discussions, parents were informed about options of fetal heart monitoring during labour, mode of delivery, primary palliative care measures, and all available treatment options. In line with our institutional policy and established ethical principles, invasive surgical procedures or intensive interventions such as intubation and mechanical ventilation were not offered. These were considered disproportionate, given the limited likelihood of meaningful benefit and the high burden, pain, and stress they would impose on infants with trisomy 13 or 18. Clinical decision-making was therefore guided by the principle of proportionality, with emphasis on palliative measures that maximize comfort and minimize suffering for both infants and their families. Counselling was non-directive within predefined therapeutic boundaries. Based on proportionality, intubation, mechanical ventilation and high-risk major surgery were not offered. Prenatal consultations outlined prognosis and these boundaries. For liveborn infants breathing spontaneously, comfort care (feeding support, warmth, symptom relief) was provided. If stabilization without invasive measures was achieved, options such as gastric-tube feeding at home and mobile paediatric palliative-care support were offered. Potential later minor surgical procedures (e.g., surgical feeding-tube placement, pulmonary artery banding or ductal stenting) were discussed only in the event of sustained stability.

Pregnancy outcomes were recorded and categorised as miscarriage (MC) before 20 WoP, termination of pregnancy (TOP), intrauterine fetal death (IUFD) after 20 WoP, or live birth. Cases with incomplete data sets or loss of follow-up were excluded (in our cohort 2 patients with loss of follow up were excluded). Potential selection bias was minimised by including all consecutive cases with genetically confirmed T13 or T18 during the study period.

Maternal characteristics, medical history, data on the clinical course of pregnancy, and delivery were extracted from our perinatal database (viewpoint), retrospectively. Relevant data considering the postnatal clinical course of the newborns were acquired from the electronic patient documentation system.

Ethical approval was obtained from the Ethics Committee of the Medical University of Vienna. The research was conducted in accordance with the Declaration of Helsinki.

### Statistical analysis

Metric variables were expressed as mean ± SD for normally distributed data, or as median and interquartile range (IQR) for non-normally distributed data. Comparisons of non-normally distributed data were performed using Mann–Whitney U-test. The Chi-squared test was applied to compare binary and categorical variables. Statistical significance was determined with a two-sided p-value of < 0.05. All analyses were performed using IBM SPSS Statistics version 23. All tests were two-sided; no adjustments were made for multiple comparisons due to the exploratory nature of the study.

## Results

Overall, 193 pregnancies were diagnosed with fetal T13 and T18 in a single tertiary prenatal center between 2005 and 2023. Of those, 142 (73.6%) fetuses were affected by T18 and 51 (26.4%) by T13. The mean maternal age at diagnosis was 35.2 years. There were no significant differences between patients affected by trisomy 13 compared to those expecting a fetus with trisomy 18. Patients’ characteristics are summarized and groups are compared in Table 1.

**Table 1:**
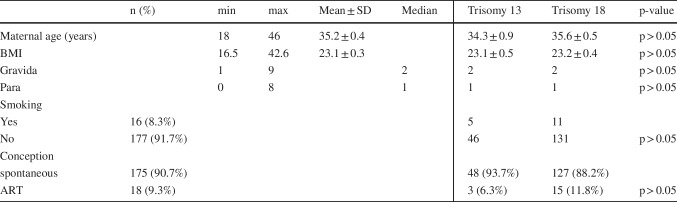
Patients characteristics

### Diagnostics

The median gestational age at diagnosis was 14.4 WoP, ranging from 11 to 36.8 WoP. The majority, of pregnant women (161, 83.4%) underwent first trimester screening between 11 + 0 and 13 + 6 WoP.

The average nuchal translucency was 4.5 mm (SD ± 0.25), with a slightly higher mean measurement in cases affected by T18 (4.63, SD ± 0.29) compared to those with T13 (4.30 mm, SD ± 0.54).

Associated malformations were found in 90.7% (175) of cases. The most commonly observed structural anomalies were cardiac malformation in 81 cases (42%). Signs of fetal hydrops were significantly more commonly found in fetuses with T18 compared to T13 (p < 0.05). Details on structural malformations and affected organ systems are summarized in Table 2.

**Table 2:** Detected malformations in children diagnosed with trisomy 13 and 18

	Trisomy 13 N (%)	Trisomy 18 N (%)	Overall
No structural malformation	5 (9.8%)	13 (9.2%)	18 (9.3%)
Cardiac malformation	22 (43.1%)	59 (41.5%)	81 (42.0%)
CNS malformation	20 (39.3%)	43 (30.3%)	63 (32.6%)
GIT malformation	14 (27.5%)	44 (31.0%)	58 (30.1%)
CAKUT	8 (15.7%)	7 (4.9%)	15 (7.8%)
Skeletal malformation	12 (23.5%)	46(32.4%)	58 (30.1%)
Facial/Scull malformation	17 (33.3%)	29 (20.4%)	46 (23.8%)
Signs of hydrops	4 (8.2%)	33 (23.2%)	37 (19.4%)

CNS malformation: central nervous system malformation; GIT malformation: Gastro-intestinal malformation; CAKUT: congenital anomalies of the Kidney and urinary tract

### Outcome

#### Termination of pregnancy

Overall, 161 (83,4%) pregnant women opted for termination of pregnancies (TOP), including 86.3% of pregnancies affected by T13 and 82.4% of those affected by T18. The median gestational age at diagnosis was significantly higher among women opting to continue the pregnancy (22.0 WoP) compared to those who opted for termination (14.1 WoP).

Nevertheless, 10.6% of TOPs had additional feticide, with a median gestational age at diagnosis of 23.3 WoP. The median number of associated malformations was comparable between groups, with a median of two malformations diagnosed in each fetus.

**Fig. 1:**
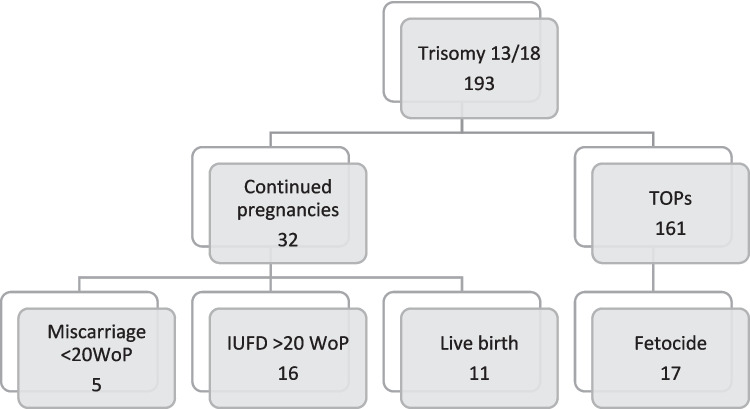
Pregnancy Outcome of fetuses diagnosed with trisomy 13 and 18

In the group of women deciding to continue pregnancy, the majority of fetuses (65.6%; 21/32) died during the course of pregnancy (Fig. 1). Whilst only 2 of 7 fetuses diagnosed with T13 died in utero, the vast majority of fetuses with T18 resulted in miscarriage or intrauterine fetal death (76%; 19/25).

In the group of patients who chose to continue pregnancy, adverse outcomes were not associated with the number of structural malformations. None of the 11 liveborn fetuses had signs of hydrops.

**Fig. 2:**
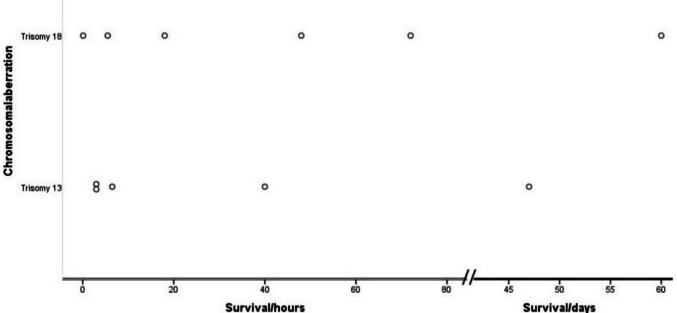
Survival length of patients affected by trisomy 13 and 18

The median gestational age at birth was 36.3 WoP, ranging from 33 to 40.1 WoP. The length of survival in life born fetuses ranged from 6 min to a maximum of 60 days, with a median survival time of 18 h. Two children, one with T18 and one with T13, survived as long as 60 and 47 days, respectively. Newborns affected by T18 had a significantly longer median survival (33 h) compared to those affected by T13 (6.5 h). The length of survival of each individual patient is depictured in Fig. 2. Whereas three children received a gastric feeding tube, none of the newborns underwent surgical intervention. Eight of eleven affected children died during their mother´s hospital stay. One woman opted for an outpatient delivery and went to a children’s hospice with her newborn, where the child died three days later. The two children who survived for more than a month were initially cared for at home after discharge, with support from a mobile paediatric palliative care team in one case. However, both were later readmitted to hospital, where they died as inpatients.

## Discussion

This study provides a comprehensive analysis of pregnancies diagnosed with T13 and T18 over an 18-year period in a single tertiary prenatal center, emphasizing prenatal and postnatal management challenges associated with these severe chromosomal conditions.

The study highlights the important role of early prenatal screening in the diagnosis of T13 and T18, with 83.4% of cases diagnosed in the first trimester. In total, 161 (83,4%) pregnant women opted for termination of pregnancy after receiving the diagnosis of T18 or T13, including 86.3% of pregnancies affected by T13 and 82.4% of those affected by T18. Notably, the median gestational age at diagnosis was significantly higher among women opting to continue pregnancy, suggesting that later diagnoses may influence decision-making and outcomes. Not only gestational age at diagnosis, but also the severity of associated anomalies influenced prenatal decision-making. A study, not specifically focused on trisomy 13 and 18, supports our findings. Tan et al. described the clinical course and outcome of fetuses with prenatally detected central nervous system anomalies and analysed parental decisions regarding termination or continuation of pregnancy. They found that around 62% of parents opted for termination when the diagnosis was made before 24 WoP, whereas after reaching viability, up to 85% chose to continue the pregnancy [7]. Furthermore, the presence of chromosomal abnormalities significanlty influenced parental decision-making, while maternal age and previous pregnancies did not [7]. Overall, various factors contribute to the decision-making process, some of which are not purely based on rational considerations [8, 9]. For pregnancies continued beyond diagnosis, comprehensive interdisciplinary care – including maternal–fetal medicine specialists, neonatologists, pediatric palliative care teams, nursing staff, and psychosocial support services – plays a crucial role. Prenatal consultations should focus not only on the medical prognosis, but also on preparing families for potential neonatal outcomes, including the likelihood of intrauterine fetal demise (IUFD) or short neonatal survival. Joint discussions between clinicians and expecting parents have been shown to support families in making informed decisions. However, the process should remain non-directive, with medical professionals adopting a neutral position to uphold the autonomy of the couple [10]. In our center, all patients facing such a diagnosis, whether they choose to continue the pregnancy or not, are offered a consultation with neonatologists to discuss the prognosis, possible interventions, and comfort care options. In addition, we offer a dedicated interdisciplinary palliative care consultation, involving maternal–fetal medicine specialists, psychologists, pediatric palliative care specialists, and often social workers. A joint approach is established on an individual basis, respecting the autonomy and wishes of the parents while acknowledging the severity of the diagnosis.

### Postnatal Management and Outcomes

The postnatal management of neonates with T13 and T18 presents unique challenges due to their usually short survival and the complexity of associated anomalies. In this study, the median survival time for live-born neonates was 18 h, with longer survival observed in T18 compared to T13. In total, the length of survival in life born infants ranged from 6 min to a maximum of 60 days. Two infants, one with T18 and one affected by T13, survived as long as 60 and 47 days, respectively. These results are consistent with prior reports describing survival intervals for T13 and T18 with 7 to 10 days and 10 to 14.5 days, respectively [2, 11]. Meyer et al. investigated survival of 1,113 children with T18 and 693 children with T13. Median survival for children with T18 and T13 was 8 and 5 days, respectively.

Both chromosomal aberrations were associated with a very high risk of early mortality, with better survival observed among children who received more aggressive medical interventions [4]. In contrast, Subramaniam et al. evaluated the clinical course and outcomes of T18 cases over a 10-year observation period and found no difference in survival and survival time between groups receiving aggressive versus non-aggressive treatment. Aggressive intervention was defined as measures intended to prolong life, including corrective surgical procedures, gastrostomy tube placement, provision of parenteral nutrition, cardiopulmonary resuscitation, and mechanical ventilation [12]. Both articles address a critical and timely question: the impact of medical and surgical interventions on the outcomes of newborns and infants with T18 or T13. In this context, Kosho and Carey questioned whether the differing results between the two studies might be explained by an oversimplified definition of aggressive and non-aggressive management [13]. A more holistic approach might be needed to evaluate which interventions may increase survival, considering not only specific procedures (such as cesarean delivery, mechanical ventilation, and cardiac surgery) but also broader supportive strategies within intensive management. Another recent study examined this question in greater detail, analysing the clinical courses and outcomes of 467 children with T13 and T18 who were admitted to a neonatal intensive care unit and received medical and surgical interventions within a large multi-center collaboration between 2010 and 2016 [14]. It was shown that 27% of the infants received surgical interventions, most commonly gastrostomy tube placement, and 62% received intensive medical treatment. Overall, discharge from the hospital was possible in 40% of these infants, with the majority referred to palliative care. [14]. A recent population-based study from Cincinnati highlighted the high healthcare burden and prolonged hospital stays among infants with trisomy 13 and 18, showing that although nearly 20% survived beyond one year, many required extensive medical support, repeated surgeries, and multiple or prolonged hospitalisations. Overall, the length of survival was highly variable and could not be reliably predicted [15]. These findings underline the importance of an individualised approach to neonatal care, carefully weighing the options between palliative care and more invasive interventions. At the same time, they also highlight the significant burden placed on infants and families, raising important ethical considerations about offering intensive care and surgical interventions aimed solely at prolonging life without sufficiently addressing the quality of life for the affected infants and their families. Hence, palliative care plays a central role in the postnatal management of affected neonates, ensuring comfort and dignity during the limited survival period. In our cohort, primary palliative care measures, including pain management, feeding support through gastric tubes, and family-centered care, were widely employed. Importantly, palliative care was initiated prenatally, with discussions on delivery planning, resuscitation preferences, and neonatal interventions offered through a neonatal consultation, ensuring alignment with the family´s goals and values. Our approach to counselling was non-directive but not neutral. Rather than presenting all conceivable treatments, we provided guidance within ethically defined limits reflecting proportionality, whereby invasive intensive care (intubation, mechanical ventilation) and high-risk major surgery were not available options given their unfavorable balance of burden and likely benefit. Parents were informed of these boundaries and supported in shared decision-making regarding comfort-focused care and, where feasible after non-invasive stabilization, home-based palliative care (gastric-tube feeding) with community support. Minor palliative procedures were considered only if stability persisted over time, however, none were undertaken in our cohort.

While three infants were supported with a gastric feeding tube, none of the newborn underwent surgery. Regarding mode of delivery, a planned cesarean section was performed in four cases. However, in two of these cases, the decision was less related to the affected child, as both women were carrying twins with only one twin diagnosed with T13 or T18. Another woman underwent cesarean section due to a history of previous cesarean delivery, and the fourth patient required cesarean delivery because of preeclampsia.

On the other hand, seven women had vaginal deliveries. Only one requested CTG monitoring with the option of cesarean section in case of fetal distress. One woman opted for an outpatient delivery and went to a children’s hospice where her newborn died three days later. Of the two neonates surviving beyond one month, both were discharged home, with one receiving support from a pediatric palliative care team. This demonstrates the potential for some families to experience meaningful time with their child, even in the context of a terminal diagnosis. Strengthening home-based palliative care services and integrating hospice care options can significantly enhance the quality of life for affected families.

### Implications for clinical practice

Despite the poor prognosis associated with T13 and T18, the study highlights the importance of individualized care strategies. It underscores the need for a multidisciplinary approach to managing affected pregnancies, involving maternal–fetal medicine specialists, neonatologists, pediatric palliative care teams, and psychosocial support. As based on the evidence in the literature, an increasing number of parents are being offered and are choosing medical interventions despite the limited prognosis [16]. At our centre, however, in line with an institutional policy aimed at minimising burden, pain, and stress for these vulnerable patients, we have not offered early invasive surgical procedures or intensive interventions such as intubation and mechanical ventilation so far. This approach is clearly communicated to families, alongside the offer of a comprehensive individualised support to ensure the child’s comfort and dignity. Counselling includes discussions on delivery planning, place of birth, and the available neonatal support options, focusing on measures to provide comfort and facilitate, where possible, discharge home with care by an outpatient palliative care team. Interventions such as feeding tube placement are offered when aligned with the goal of maximising comfort and quality of life. For pregnancies continued to term, close coordination with neonatal teams ensures a seamless transition from prenatal to postnatal care.

### Strengths of the study

A major strength of this study is the large sample size of 193 genetically confirmed cases of T13 and T18 collected over an 18-year period**.**This long period allows for a comprehensive evaluation of trends in prenatal diagnostics, parental decision-making, and perinatal as well as postnatal management strategies.

Additionally, the study benefits from a highly standardised diagnostic work-up including detailed anomaly scans, genetic testing for confirmation, and systematic follow-up.

### Limitations of the study

This study also has some limitations that should be considered when interpreting the findings. First, its retrospective and single-centre design may limit the generalizability of the results to other populations or healthcare settings with different management strategies.

Second, although the overall sample size is relatively large for such rare chromosomal conditions, the number of live-born infants remains small, which may reduce the statistical power to detect differences in neonatal outcomes and survival times. Finally, we cannot exclude residual confounding and the potential impact of unmeasured variables, such as parental psychosocial factors and cultural influences, on decision-making and neonatal outcomes.

## Conclusion

Trisomy 13 and 18 remain associated with significant structural malformations and poor outcomes, despite advances in prenatal diagnostics. Early detection and comprehensive counselling, and multidisciplinary care are essential to support informed decisions-making. Although increasing evidence suggests that some infants may experience prolonged survival with intensive-care management, counselling must carefully balance these findings against poor overall prognosis and the considerable burden of invasive interventions. In our institution, in accordance with an established care policy, invasive ventilation and early surgical procedures have not been offered so far, with postnatal management focused on comfort, quality of life, and family-centred palliative support.

## Supplementary Information

Below is the link to the electronic supplementary material.

Supplementary file1 (DOC 85 KB)

## Data Availability

The data presented in this study are available on request from the corresponding author. The data are not publicly available due to data privacy.
