# Supplementary material for: Clinical course and perinatal management of fetuses and newborns affected by trisomy 13 and 18: a retrospective single-centre cohort study
Source: Eur J Pediatr. 2025 Dec 20;185(1):30. doi: 10.1007/s00431-025-06614-7 (PMC12718251; doi:10.1007/s00431-025-06614-7)
Supplement: Supplementary file 1 — Supplementary file1 (DOC 85 KB) [file 431_2025_6614_MOESM1_ESM.doc]

STROBE Statement—Checklist of items that should be included in reports of ***cohort studies***

|  | Item No | Recommendation |
| --- | --- | --- |
| **Title and abstract**  **p 1-2** | 1 | (*a*) Indicate the study’s design with a commonly used term in the title or the abstract p1; Line 3-4 |
|  |  | (*b*) Provide in the abstract an informative and balanced summary of what was done and what was found p2; Line 40 - 64 |
| Introduction p 3-4 | | |
| Background/rationale | 2 | Explain the scientific background and rationale for the investigation being reported p3, Line 68-78 |
| Objectives | 3 | State specific objectives, including any prespecified hypotheses p3-4, Line 89-95 |
| Methods p4-6 | | |
| Study design | 4 | Present key elements of study design early in the paper p 4, Line 99-100 |
| Setting | 5 | Describe the setting, locations, and relevant dates, including periods of recruitment, exposure, follow-up, and data collection p 4, Line 100-106; 134-136 |
| Participants | 6 | (*a*) Give the eligibility criteria, and the sources and methods of selection of participants. Describe methods of follow-up p 4; Line104-107; 136-144 |
|  |  | (*b*) For matched studies, give matching criteria and number of exposed and unexposed *not applicable* |
| Variables | 7 | Clearly define all outcomes, exposures, predictors, potential confounders, and effect modifiers. Give diagnostic criteria, if applicable p5 ; Line 134-139 |
| Data sources/ measurement | 8* | For each variable of interest, give sources of data and details of methods of assessment (measurement). p6, Line 141-144 Describe comparability of assessment methods if there is more than one group *not applicable- retrospective analysis* |
| Bias | 9 | Describe any efforts to address potential sources of bias p6; Line 137-139 |
| Study size | 10 | Explain how the study size was arrived at p4; Line 104-106 |
| Quantitative variables | 11 | Explain how quantitative variables were handled in the analyses. If applicable, describe which groupings were chosen and why *not applicable -retrospective design* |
| Statistical methods | 12 | (*a*) Describe all statistical methods, including those used to control for confounding p6, Line 148-155 |
|  |  | (*b*) Describe any methods used to examine subgroups and interactions |
|  |  | (*c*) Explain how missing data were addressed p5; Line 136-137 |
|  |  | (*d*) If applicable, explain how loss to follow-up was addressed p5; Line 136-137 |
|  |  | (*e*) Describe any sensitivity analyses *not applicable* |
| Results p7-10 | | |
| Participants | 13* | (a) Report numbers of individuals at each stage of study—eg numbers potentially eligible, examined for eligibility, confirmed eligible, included in the study, completing follow-up, and analysed p7; Line 164-166, p8 Line 190-191, p9 Line 199-202 |
|  |  | (b) Give reasons for non-participation at each stage *not applicable* |
|  |  | (c) Consider use of a flow diagram p9, Line 203-204 |
| Descriptive data | 14* | (a) Give characteristics of study participants (eg demographic, clinical, social) and information on exposures and potential confounders p7, Line 166-169, table1 |
|  |  | (b) Indicate number of participants with missing data for each variable of interest – not applicable - patients were excluded (Line 136-137) |
|  |  | (c) Summarise follow-up time (eg, average and total amount) p9; Line 212-215 |
| Outcome data | 15* | Report numbers of outcome events or summary measures over time p9-10 Line 207-221 |
| Main results **p7-10** | 16 | (*a*) Give unadjusted estimates and, if applicable, confounder-adjusted estimates and their precision (eg, 95% confidence interval). Make clear which confounders were adjusted for and why they were included p7-9 |
|  |  | (*b*) Report category boundaries when continuous variables were categorized *“not applicable”* |
|  |  | (*c*) If relevant, consider translating estimates of relative risk into absolute risk for a meaningful time period *“not applicable”* |
| Other analyses | 17 | Report other analyses done—eg analyses of subgroups and interactions, and sensitivity analyses p8, Line 176 – 185, p9 Line 192-194 |
| Discussion p11-17 | | |
| Key results | 18 | Summarise key results with reference to study objectives p11, Line 231-238 |
| Limitations | 19 | Discuss limitations of the study, taking into account sources of potential bias or imprecision. Discuss both direction and magnitude of any potential bias p 16/17 Line 369-379 |
| Interpretation | 20 | Give a cautious overall interpretation of results considering objectives, limitations, multiplicity of analyses, results from similar studies, and other relevant evidence p17, Line 382-391 |
| Generalisability | 21 | Discuss the generalisability (external validity) of the study results p17, Line 392-395 |
| Other information | | |
| Funding | 22 | Give the source of funding and the role of the funders for the present study and, if applicable, for the original study on which the present article is based p1 Line 33 |

*Give information separately for exposed and unexposed groups.

**Note:** An Explanation and Elaboration article discusses each checklist item and gives methodological background and published examples of transparent reporting. The STROBE checklist is best used in conjunction with this article (freely available on the Web sites of PLoS Medicine at http://www.plosmedicine.org/, Annals of Internal Medicine at http://www.annals.org/, and Epidemiology at http://www.epidem.com/). Information on the STROBE Initiative is available at http://www.strobe-statement.org.
